# 3,5-Dihydr­oxy-*N*′-(2-hydroxy­benzyl­idene)benzohydrazide monohydrate

**DOI:** 10.1107/S1600536807067177

**Published:** 2007-12-21

**Authors:** Qing-Hua Jiang, Ying-Hong Xu, Ling-Yan Jian, Li-Mei Zhao

**Affiliations:** a Department of Pharmacy, Affiliated Shengjing Hospital, China Medical University, Shenyang 110004, People’s Republic of China

## Abstract

The title potential anti­bacterial compound, C_14_H_12_N_2_O_4_·H_2_O, is a Schiff base which has an intra­molecular O—H⋯N hydrogen bond and crystallizes with one uncoordinated water mol­ecule, which links three symmetry-related mol­ecules through two O—H⋯O and one N—H⋯O hydrogen bond. In the crystal structure, further inter­molecular O—H⋯O hydrogen bonds link symmetry-related mol­ecules, forming layers parallel to the *bc* plane.

## Related literature

For related structures, see: Ali *et al.* (2005 ▶); Diao (2007 ▶); Diao, Li *et al.* (2007 ▶); Diao, Shu *et al.* (2007 ▶); Diao, Wang *et al.* (2007 ▶); Jing *et al.* (2006 ▶); Qiu *et al.* (2006 ▶); Wang *et al.* (2007 ▶); Yang (2007 ▶).
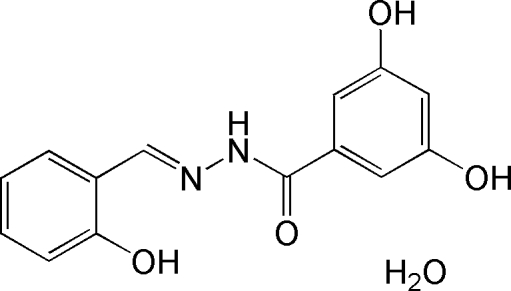


## Experimental

### 

#### Crystal data

C_14_H_12_N_2_O_4_·H_2_O
*M* *_r_* = 290.27
Monoclinic, 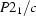

*a* = 7.773 (2) Å
*b* = 13.411 (3) Å
*c* = 13.084 (3) Å
β = 100.52 (3)°
*V* = 1341.0 (5) Å^3^
*Z* = 4
Mo *K*α radiation
μ = 0.11 mm^−1^
*T* = 293 (2) K
0.33 × 0.32 × 0.32 mm

#### Data collection

Bruker SMART APEX area-detector diffractometer
Absorption correction: multi-scan (*SADABS*; Sheldrick, 1996 ▶) *T*_min_ = 0.964, *T*_max_ = 0.965
17195 measured reflections
2918 independent reflections
2062 reflections with *I* > 2σ(*I*)
*R*_int_ = 0.038

#### Refinement

*R*[*F* ^2^ > 2σ(*F* ^2^)] = 0.045
*wR*(*F* ^2^) = 0.125
*S* = 1.03
2918 reflections
202 parameters
4 restraints
H atoms treated by a mixture of independent and constrained refinement
Δρ_max_ = 0.56 e Å^−3^
Δρ_min_ = −0.21 e Å^−3^

### 

Data collection: *SMART* (Siemens, 1996 ▶); cell refinement: *SAINT* (Siemens, 1996 ▶); data reduction: *SAINT*; program(s) used to solve structure: *SHELXS97* (Sheldrick, 1997*a* ▶); program(s) used to refine structure: *SHELXL97* (Sheldrick, 1997*a* ▶); molecular graphics: *SHELXTL* (Sheldrick, 1997*b* ▶); software used to prepare material for publication: *SHELXTL*.

## Supplementary Material

Crystal structure: contains datablocks global, I. DOI: 10.1107/S1600536807067177/su2036sup1.cif

Structure factors: contains datablocks I. DOI: 10.1107/S1600536807067177/su2036Isup2.hkl

Additional supplementary materials:  crystallographic information; 3D view; checkCIF report

## Figures and Tables

**Table 1:** Hydrogen-bond geometry (Å, °)

*D*—H⋯*A*	*D*—H	H⋯*A*	*D*⋯*A*	*D*—H⋯*A*
O1—H1⋯N1	0.82	1.86	2.587 (2)	147
O5—H5*B*⋯O2	0.857 (9)	1.96 (1)	2.807 (2)	170 (2)
O5—H5*A*⋯O3^i^	0.856 (9)	1.96 (1)	2.806 (2)	168 (2)
N2—H2*A*⋯O5^ii^	0.899 (10)	1.96 (1)	2.852 (2)	170 (2)
O3—H3⋯O2^ii^	0.82	1.87	2.682 (2)	173
O4—H4⋯O1^iii^	0.82	2.00	2.813 (2)	169

Symmetry codes: (i) 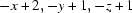; (ii) 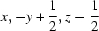; (iii) 
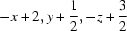
.

**Fig. 1.**
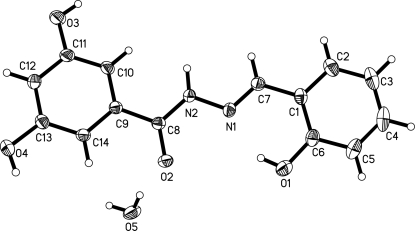
The molecular structure of the title compound, showing the numbering scheme and displacement ellipsoids drawn at the 30% probability level

**Fig. 2.**
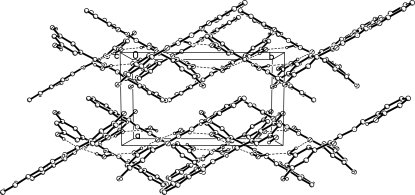
The crystal packing of the the title compound. The intermolecular hydrogen bonds are shown as dashed lines.

## supplementary crystallographic information

### Comment

Compounds derived from the Schiff base condensation reaction of aldehydes with hydrazides have been widely investigated both from a structural point of view and for their biological activity (Jing *et al.*, 2006; Yang, 2007; Wang *et al.*, 2007). Complexes derived from Schiff bases have also been widely investigated (Ali *et al.*, 2005; Qiu *et al.*, 2006; Diao, 2007; Diao, 2007; Diao, Li *et al.*, 2007; Diao, Shu *et al.*, 2007; Diao, Wang *et al.*, 2007). We report herein the crystal structure of the title compound derived from the reaction of equimolar salicylaldehyde with 3,5-dihydroxybenzoic acid hydrazide in a methanol solution.

The molecular structure of the title compound (Fig. 1) is a Schiff base, which has an intramolecular O1—H1···N1 hydrogen bond (Table 1), and crystallizes as a water solvate. In the crystal structure the water molecule links three symmetry related molecules through two donnor O—H···O hydrogen bonds and one acceptor N—H···O hydrogen bond (Table 1). Together with two further intermolecular O—H···O hydrogen bonds, layers parallel to the *bc* plane are formed (Fig. 2).

### Experimental

Salicylaldehyde and 3,5-dihydroxybenzoic acid hydrazide were purchased from Aldrich and were used without further purification. Salicylaldehyde (0.1 mmol, 12.2 mg) and 3,5-dihydroxybenzoic acid hydrazide (0.1 mmol, 16.8 mg) were mixed in a methanol solution (10 cm^3^). The mixture was stirred at reflux for 30 min and cooled to room temperature. After keeping the solution in air for a few days, yellow block-shaped crystals appeard at the bottom of the vessel.

### Refinement

The NH H-atom, H2A, and the water H-atoms were located from difference Fourier maps and were refined with the N–H, O–H and H···H distances restrained to 0.90 (1), 0.85 (1) and 1.37 (2) Å, respectively. The remaining H-atoms were placed in calculated positions and treated as riding atoms; C–H = 0.93 Å with *U*_iso_(H) = 1.2*U*_eq_(C), and O–H = 0.82 Å with *U*_iso_(H) = 1.5*U*_eq_(O).

### Crystal data

**Table:**

C_14_H_12_N_2_O_4_·H_2_O	*F*_000_ = 608
*M**_r_* = 290.27	*D*_x_ = 1.438 Mg m^−^^3^
Monoclinic, *P*2_1_/*c*	Mo *K*α radiation λ = 0.71073 Å
Hall symbol: P 2ybc	Cell parameters from 2799 reflections
*a* = 7.773 (2) Å	θ = 2.2–24.5º
*b* = 13.411 (3) Å	µ = 0.11 mm^−^^1^
*c* = 13.084 (3) Å	*T* = 293 (2) K
β = 100.52 (3)º	Block, yellow
*V* = 1341.0 (5) Å^3^	0.33 × 0.32 × 0.32 mm
*Z* = 4	

### Data collection

**Table:**

Bruker SMART APEX area-detector diffractometer	2918 independent reflections
Radiation source: fine-focus sealed tube	2062 reflections with *I* > 2σ(*I*)
Monochromator: graphite	*R*_int_ = 0.038
*T* = 293(2) K	θ_max_ = 27.0º
ω scans	θ_min_ = 2.2º
Absorption correction: multi-scan(SADABS; Sheldrick, 1996)	*h* = −9→9
*T*_min_ = 0.964, *T*_max_ = 0.965	*k* = −16→17
17195 measured reflections	*l* = −16→16

### Refinement

**Table:**

Refinement on *F*^2^	Secondary atom site location: difference Fourier map
Least-squares matrix: full	Hydrogen site location: inferred from neighbouring sites
*R*[*F*^2^ > 2σ(*F*^2^)] = 0.045	H atoms treated by a mixture of independent and constrained refinement
*wR*(*F*^2^) = 0.125	*w* = 1/[σ^2^(*F*_o_^2^) + (0.0542*P*)^2^ + 0.4645*P*] where *P* = (*F*_o_^2^ + 2*F*_c_^2^)/3
*S* = 1.03	(Δ/σ)_max_ < 0.001
2918 reflections	Δρ_max_ = 0.56 e Å^−^^3^
202 parameters	Δρ_min_ = −0.21 e Å^−^^3^
4 restraints	Extinction correction: none
Primary atom site location: structure-invariant direct methods	

### Special details

**Table:**

Geometry. All e.s.d.'s (except the e.s.d. in the dihedral angle between two l.s. planes) are estimated using the full covariance matrix. The cell e.s.d.'s are taken into account individually in the estimation of e.s.d.'s in distances, angles and torsion angles; correlations between e.s.d.'s in cell parameters are only used when they are defined by crystal symmetry. An approximate (isotropic) treatment of cell e.s.d.'s is used for estimating e.s.d.'s involving l.s. planes.
Refinement. Refinement of *F*^2^ against ALL reflections. The weighted *R*-factor *wR* and goodness of fit *S* are based on *F*^2^, conventional *R*-factors *R* are based on *F*, with *F* set to zero for negative *F*^2^. The threshold expression of *F*^2^ > σ(*F*^2^) is used only for calculating *R*-factors(gt) *etc*. and is not relevant to the choice of reflections for refinement. *R*-factors based on *F*^2^ are statistically about twice as large as those based on *F*, and *R*- factors based on ALL data will be even larger.

### Fractional atomic coordinates and isotropic or equivalent isotropic displacement parameters (Å^2^)

**Table:**

	*x*	*y*	*z*	*U*_iso_*/*U*_eq_	
N1	0.82125 (17)	0.06536 (10)	0.52477 (11)	0.0340 (3)	
N2	0.89934 (18)	0.14758 (10)	0.48954 (11)	0.0329 (3)	
O1	0.7166 (2)	−0.03870 (11)	0.66833 (10)	0.0593 (4)	
H1	0.7590	0.0093	0.6430	0.089*	
O2	0.96488 (18)	0.20688 (9)	0.65336 (9)	0.0452 (3)	
O3	1.1037 (3)	0.43849 (11)	0.28345 (10)	0.0700 (5)	
H3	1.0537	0.3942	0.2462	0.105*	
O4	1.3445 (2)	0.50690 (11)	0.63155 (11)	0.0577 (4)	
H4	1.3389	0.4896	0.6910	0.087*	
O5	0.8486 (2)	0.36158 (10)	0.76830 (11)	0.0606 (4)	
C1	0.6575 (2)	−0.08377 (12)	0.48772 (14)	0.0369 (4)	
C2	0.5775 (2)	−0.15150 (14)	0.41236 (17)	0.0508 (5)	
H2	0.5807	−0.1397	0.3427	0.061*	
C3	0.4943 (3)	−0.23513 (15)	0.4397 (2)	0.0613 (6)	
H3A	0.4418	−0.2794	0.3887	0.074*	
C4	0.4886 (3)	−0.25336 (15)	0.5423 (2)	0.0635 (7)	
H4A	0.4340	−0.3106	0.5607	0.076*	
C5	0.5630 (3)	−0.18744 (16)	0.61818 (19)	0.0579 (6)	
H5	0.5571	−0.1997	0.6874	0.070*	
C6	0.6470 (2)	−0.10272 (13)	0.59157 (15)	0.0424 (4)	
C7	0.7438 (2)	0.00390 (12)	0.45641 (14)	0.0363 (4)	
H7	0.7431	0.0157	0.3863	0.044*	
C8	0.9672 (2)	0.21721 (12)	0.55913 (12)	0.0319 (4)	
C9	1.0484 (2)	0.30675 (11)	0.51996 (12)	0.0315 (4)	
C10	1.0290 (2)	0.33013 (13)	0.41515 (13)	0.0399 (4)	
H10	0.9576	0.2916	0.3656	0.048*	
C11	1.1174 (3)	0.41156 (13)	0.38544 (14)	0.0431 (4)	
C12	1.2233 (3)	0.46968 (13)	0.45862 (14)	0.0424 (4)	
H12	1.2839	0.5235	0.4378	0.051*	
C13	1.2382 (2)	0.44731 (13)	0.56219 (13)	0.0385 (4)	
C14	1.1516 (2)	0.36597 (12)	0.59365 (13)	0.0354 (4)	
H14	1.1625	0.3511	0.6640	0.042*	
H2A	0.894 (3)	0.1496 (18)	0.4203 (8)	0.080*	
H5A	0.876 (3)	0.4197 (9)	0.7493 (18)	0.080*	
H5B	0.885 (3)	0.3199 (13)	0.7274 (16)	0.080*	

### Atomic displacement parameters (Å^2^)

**Table:**

	*U*^11^	*U*^22^	*U*^33^	*U*^12^	*U*^13^	*U*^23^
N1	0.0361 (7)	0.0282 (7)	0.0391 (8)	−0.0005 (6)	0.0105 (6)	0.0015 (6)
N2	0.0397 (8)	0.0282 (7)	0.0319 (7)	−0.0035 (6)	0.0099 (6)	−0.0004 (6)
O1	0.0736 (10)	0.0606 (9)	0.0411 (8)	−0.0153 (8)	0.0033 (7)	0.0109 (7)
O2	0.0685 (9)	0.0388 (7)	0.0287 (7)	−0.0058 (6)	0.0104 (6)	0.0020 (5)
O3	0.1327 (15)	0.0443 (8)	0.0319 (7)	−0.0293 (9)	0.0125 (8)	0.0041 (6)
O4	0.0694 (9)	0.0560 (9)	0.0443 (8)	−0.0272 (7)	0.0015 (7)	−0.0049 (7)
O5	0.1062 (13)	0.0392 (8)	0.0432 (8)	0.0067 (8)	0.0312 (8)	0.0064 (6)
C1	0.0316 (8)	0.0285 (8)	0.0517 (11)	0.0031 (7)	0.0101 (8)	−0.0023 (7)
C2	0.0445 (10)	0.0444 (11)	0.0654 (13)	−0.0034 (9)	0.0149 (9)	−0.0156 (10)
C3	0.0442 (11)	0.0385 (11)	0.102 (2)	−0.0061 (9)	0.0148 (12)	−0.0205 (12)
C4	0.0443 (11)	0.0326 (10)	0.115 (2)	−0.0027 (9)	0.0177 (12)	0.0101 (12)
C5	0.0530 (12)	0.0481 (12)	0.0726 (15)	−0.0013 (10)	0.0110 (11)	0.0226 (11)
C6	0.0397 (10)	0.0336 (9)	0.0524 (12)	0.0017 (7)	0.0043 (8)	0.0086 (8)
C7	0.0385 (9)	0.0353 (9)	0.0371 (10)	0.0010 (7)	0.0117 (7)	−0.0017 (7)
C8	0.0366 (9)	0.0299 (8)	0.0292 (9)	0.0037 (7)	0.0061 (7)	0.0010 (7)
C9	0.0375 (9)	0.0271 (8)	0.0303 (9)	0.0026 (7)	0.0071 (7)	−0.0004 (6)
C10	0.0586 (11)	0.0287 (8)	0.0304 (9)	−0.0043 (8)	0.0030 (8)	−0.0008 (7)
C11	0.0684 (12)	0.0312 (9)	0.0305 (9)	−0.0030 (8)	0.0108 (8)	0.0017 (7)
C12	0.0562 (11)	0.0305 (9)	0.0418 (11)	−0.0067 (8)	0.0121 (8)	0.0011 (8)
C13	0.0425 (10)	0.0340 (9)	0.0382 (10)	−0.0030 (7)	0.0056 (8)	−0.0044 (7)
C14	0.0423 (9)	0.0356 (9)	0.0281 (9)	0.0000 (7)	0.0057 (7)	−0.0004 (7)

### Geometric parameters (Å, °)

**Table:**

N1—C7	1.283 (2)	C3—C4	1.373 (3)
N1—N2	1.3780 (19)	C3—H3A	0.9300
N2—C8	1.343 (2)	C4—C5	1.375 (3)
N2—H2A	0.899 (10)	C4—H4A	0.9300
O1—C6	1.357 (2)	C5—C6	1.386 (3)
O1—H1	0.8200	C5—H5	0.9300
O2—C8	1.244 (2)	C7—H7	0.9300
O3—C11	1.368 (2)	C8—C9	1.490 (2)
O3—H3	0.8200	C9—C14	1.386 (2)
O4—C13	1.368 (2)	C9—C10	1.388 (2)
O4—H4	0.8200	C10—C11	1.383 (2)
O5—H5A	0.856 (9)	C10—H10	0.9300
O5—H5B	0.857 (9)	C11—C12	1.383 (3)
C1—C6	1.399 (3)	C12—C13	1.372 (3)
C1—C2	1.400 (3)	C12—H12	0.9300
C1—C7	1.450 (2)	C13—C14	1.383 (2)
C2—C3	1.374 (3)	C14—H14	0.9300
C2—H2	0.9300		
			
C7—N1—N2	117.40 (14)	C5—C6—C1	120.43 (19)
C8—N2—N1	118.17 (13)	N1—C7—C1	120.45 (16)
C8—N2—H2A	126.9 (16)	N1—C7—H7	119.8
N1—N2—H2A	114.8 (16)	C1—C7—H7	119.8
C6—O1—H1	109.5	O2—C8—N2	121.30 (15)
C11—O3—H3	109.5	O2—C8—C9	120.94 (15)
C13—O4—H4	109.5	N2—C8—C9	117.75 (14)
H5A—O5—H5B	106.6 (18)	C14—C9—C10	120.16 (15)
C6—C1—C2	117.96 (17)	C14—C9—C8	116.75 (15)
C6—C1—C7	122.26 (16)	C10—C9—C8	123.06 (15)
C2—C1—C7	119.75 (17)	C11—C10—C9	119.09 (16)
C3—C2—C1	121.0 (2)	C11—C10—H10	120.5
C3—C2—H2	119.5	C9—C10—H10	120.5
C1—C2—H2	119.5	O3—C11—C10	121.91 (17)
C4—C3—C2	120.1 (2)	O3—C11—C12	117.19 (16)
C4—C3—H3A	120.0	C10—C11—C12	120.90 (17)
C2—C3—H3A	120.0	C13—C12—C11	119.52 (17)
C3—C4—C5	120.4 (2)	C13—C12—H12	120.2
C3—C4—H4A	119.8	C11—C12—H12	120.2
C5—C4—H4A	119.8	O4—C13—C12	117.35 (16)
C4—C5—C6	120.1 (2)	O4—C13—C14	122.08 (16)
C4—C5—H5	120.0	C12—C13—C14	120.54 (16)
C6—C5—H5	120.0	C13—C14—C9	119.75 (16)
O1—C6—C5	118.38 (18)	C13—C14—H14	120.1
O1—C6—C1	121.19 (16)	C9—C14—H14	120.1

### Hydrogen-bond geometry (Å, °)

**Table:**

*D*—H···*A*	*D*—H	H···*A*	*D*···*A*	*D*—H···*A*
O1—H1···N1	0.82	1.86	2.587 (2)	147
O5—H5B···O2	0.857 (9)	1.96 (1)	2.807 (2)	170 (2)
O5—H5A···O3^i^	0.856 (9)	1.96 (1)	2.806 (2)	168 (2)
N2—H2A···O5^ii^	0.899 (10)	1.96 (1)	2.852 (2)	170 (2)
O3—H3···O2^ii^	0.82	1.87	2.682 (2)	173
O4—H4···O1^iii^	0.82	2.00	2.813 (2)	169

Symmetry codes: (i) −*x*+2, −*y*+1, −*z*+1; (ii) *x*, −*y*+1/2, *z*−1/2; (iii) −*x*+2, *y*+1/2, −*z*+3/2.
